# Detection of antibiotic resistant *Acinetobacter baumannii* in various hospital environments: potential sources for transmission of *Acinetobacter* infections

**DOI:** 10.1186/s12199-017-0653-4

**Published:** 2017-05-08

**Authors:** Zahra Shamsizadeh, Mahnaz Nikaeen, Bahram Nasr Esfahani, Seyed Hamed Mirhoseini, Maryam Hatamzadeh, Akbar Hassanzadeh

**Affiliations:** 1 Department of Environmental Health Engineering, School of Health, Isfahan University of Medical Sciences, Hezar Jerib Avenue, Isfahan, Iran; 2 Department of Microbiology, School of Medicine, Isfahan University of Medical Sciences, Isfahan, Iran; 3 Department of Environmental Health Engineering, School of Health, Arak University of Medical Sciences, Arak, Iran; 4 Department of Statistics and Epidemiology, School of Health, Isfahan University of Medical Sciences, Isfahan, Iran

**Keywords:** *Acinetobacter baumannii*, Hospital, Antibiotic resistance, Air, Water, Surface

## Abstract

**Background:**

Antibiotic resistant *Acinetobacter baumannii* has emerged as one of the most problematic hospital acquired pathogens around the world. This study was designed to investigate the presence of antibiotic resistant *A. baumannii* in various hospital environments.

**Methods:**

Air, water and inanimate surface samples were taken in different wards of four hospitals and analyzed for the presence of *A. baumannii*. Confirmed *A. baumannii* isolates were analyzed for antimicrobial susceptibility and also screened for the presence of three most common OXA- type carbapenemase-encoding genes.

**Results:**

*A. baumannii* was detected in 11% (7/64) of air samples with the highest recovery in intensive care units (ICUs). *A. baumannii* was also detected in 17% (7/42) and 2% (1/42) of surface and water samples, respectively. A total of 40 *A. baumannii* isolates were recovered and analysis of antimicrobial susceptibility showed the highest resistance towards ceftazidime (92.5%, 37/40). 85% (34/40) and 80% (32/40) of the isolates were also resistant to imipenem and gentamicin, respectively. Resistance genes analysis showed that 77.5% (31/40) strains contained OXA-23 and 5% (2/40) strains contained OXA-24, but OXA-58 was not detected in any of the strains.

**Conclusion:**

Detection of antibiotic resistant *A. baumannii* in various samples revealed that hospital environments could act as a potential source for transmission of *A. baumannii* infections especially in ICUs. These results emphasize the importance of early detection and implementation of control measures to prevent the spread of *A. baumannii* in hospital environments.

## Background

Nosocomial infections have become increasingly a major health concern in many hospitals worldwide [1, 2]. Nosocomial infections account for about 1.4 million infections every year [3]. *Acinetobacter* infections have frequently been reported as a major of nosocomial infections [1, 4, 5].

*Acinetobacter* species, ubiquitous gram-negative coccobacilli, are widespread in nature, water and soil [5, 6]. More than 20 species of *Acinetobacter* have been characterized but only few species including *Acinetobacter baumannii*, *A. calcoaceticus* and *A. lwoffii* play a significant role in nosocomial infections [6]. However, *A. baumannii* has the greatest clinical significance and identified as the causative agent of the majority of nosocomial infections especially in intensive care units (ICU) [6–8]. *A. baumannii* can cause a wide range of infections including bacteremia, meningitis, urinary tract, bloodstream or surgical wound infections and ventilator associated pneumonia [5, 6].

However, the emergence of antibiotics-resistant *A. baumannii* especially, multiresistant strains seriously challenges the treatment of these infections [9]. This is of special concern in developing countries, since antibiotic prescription rates and intake without prescription is markedly higher [9]. Antibiotic resistance causing increased morbidity, mortality, and economic impacts on health services [2]. Vulnerable groups of inpatients such as people with impaired host defenses are especially at high risk [4, 10]. *A. baumannii* has the ability to survive for long periods and could easily spread in hospital environments [5]. These traits could define its propensity for causing extended outbreaks [5, 6].

*A. baumannii* is mainly transmitted by direct contact with infected persons or indirect contact with contaminated environments. However, airborne route also plays an important role in transmission of *A. baumannii* infections in hospitals [2, 11]. Although, airborne transmission was considered as a route for acquisition of *A. baumannii* infections; there are very few studies in the field [11–14].

Prevention and control of hospital infections require knowledge about the sources and reservoirs of nosocomial infection agents [5]. In other words, identification of *A. baumannii* sources in hospital environments improves the knowledge of potential routes of *A. baumannii* transmission. Such information would also allow implementing more appropriate control policies against the spreading of *A. baumannii* infections.

Based on these premises, the present study was carried out in order to 1) determine the occurrence of *A. baumannii* in air, water and inanimate surface samples in different wards of four educational hospitals 2) evaluate the antibiotic resistance of isolated *A. baumannii* 3) Evaluate the frequency of three common OXA-type carbapenemase-encoding genes in isolated resistant bacteria 4) analyze the molecular diversity of *A. baumannii* isolates by repetitive extragenic palindromic sequence PCR (REP-PCR).

## Methods

The study was carried out from April 2014 to April 2015 in four educational hospitals of Isfahan University of Medical Sciences, Isfahan, Iran. Air, water and surface samples were taken in four locations in each hospital including operating theatres (OT), intensive care units (ICU), surgery wards (SW), and internal medicine wards (IM). Each hospital was visited 4 times and samples from various locations were taken on one single day after routine cleaning. A similar disinfection procedure was used for all hospitals. During the study period, patients, staffs, and patient attendants were present, but visitors were limited.

### Air samples

A total of 64 air samples were collected at a calibrated flow rate of 10 l/min using an all-glass impinger (AGI), containing phosphate buffer solution. Air sampling was performed at a height of 1.5 m above the ground level to simulate the breathing zone and approximately 2400 L of air was collected using portable pump from each site. Temperature and relative humidity were recorded by use of a portable weather station (Kimo) throughout the sampling periods and were about 26 ± 2.3 °C and 28% ± 5.6%, respectively.

### Surface samples

A total of 42 surface samples were taken from patient beds in SW, IM and ICU of four hospitals. Surface samples were obtained by swabbing of beds surface with a saline solution moistened cotton swab. After sampling, swab was placed into a sterile tube containing 2 mL of 0.8% salt water and was transferred to the laboratory.

### Water samples

Sampling of tap water was done in 250 ml bottles containing thiosulfate from SW, IM and ICU of four hospitals.

All samples were transferred to the laboratory in an insulated box with cooling packs and processed immediately upon arrival in the laboratory.

### Detection of *A. baumannii*

For detection of *A. baumannii* in air samples, aliquots of each impinger collection medium were plated onto blood agar and MacConkey agar after a vigorous shaking.

The saline suspension of surface samples was enriched overnight at 37 °C in brain heart infusion broth. Then, 0.3 ml of liquid broth transferred to each of the blood agar and MacConkey agar.

Water samples were filtered by membrane filtration (0.22 μm, 47 mm in diameter, Millipore) and then filters were placed on MacConkey agar plates.

All MacConkey and blood agar plates were incubated at 37 °C for 72 h. After incubation time non-repetitive colonies were isolated and confirmatory procedures by using conventional biochemical tests was performed [1]. Suspected colonies were also further verified using the *Acinetobacter* specific primer set Ac436F and Ac676r (Table 1), and *A. baumannii* identification was further confirmed by polymerase chain reaction (PCR) amplification of the inherent blaOXA-51 gene (Table 1) [12]. Confirmed *A. baumannii* isolates were analyzed for antimicrobial susceptibility and also screened for the presence of three most common OXA- type carbapenemase-encoding genes.

**Table 1:** Primers used in the study

Primers	Sequence (5' → 3')	Amplified fragment (bp)	Annealing temperature	Reference
Ac436 Ac676	TTTAAGCGAGGAGGAGG ATTCTACCATCCTCTCCC	240	56	[30]
OXA-51	F: TAATGCTTTGATCGGCCTTG R: TGGATTGCACTTCATCTTGG	353	57	[31]
OXA-23	F: GATCGGATTGGAGAACCAGA R: ATTTCTGACCGCATTTCCAT	501	54	[31]
OXA-24	F: GGTTAGTTGGCCCCCTTAAA R: AGTTGAGCGAAAAGGGGATT	246	54	[31]
OXA-58	F: AAGTATTGGGGCTTGTGCTG R: CCCCTCTGCGCTCTACATAC	599	54	[31]
REP	REP1: IIIGCGCCGICATCAGGC REP 2: ACGTCTTATCAGGCCTAC	-	43	[16]

### Antimicrobial susceptibility testing

Antimicrobial susceptibility analysis of the isolates was performed by disc diffusion method on Mueller–Hinton agar using ceftazidime (30 μg), imipenem (10 μg) and gentamicin (10 μg) according to the recommendations of the Clinical and Laboratory Standards Institute (CLSI) [15]. For quality control, standard strain of *E. coli* (ATCC 25922) was used.

### Detection of OXA-type carbapenemase-encoding genes

*A. baumannii* isolates were screened for 3 common OXA-type carbapenemase-encoding genes including blaOXA-23, blaOXA-58 and blaOXA-24 by PCR amplification with specific three sets of primers (Table 1).

### PCR amplification

A loopful of each isolate was put into 100 μl of deionized water. Then the suspension was vortexed and DNA was extracted by boiling for 15 min and centrifugation at 13,000 rpm for 10 min. Supernatant was used for PCR amplification. All PCR reactions were performed in a final volume of 25 μl containing 2.5 μl of 10x PCR buffer (2 mM MgCl_2_), 0.2 μM of each primer, 0.2 mM of each of the dNTPs, 2 units of Taq DNA polymerase, and 2 μl template DNA. The PCR cycling conditions were as follows: initial denaturation at 94 °C for 5 min, followed by 35 cycles of 45 s at 94 °C, primer annealing at varied temperatures (Table 1) for 45 s, primer extension at 72 °C for 45 s, and final extension at 72 °C for 10 min. All PCR assays included positive (*A. baumannii*, ATCC 19606) and negative controls. PCR products were analyzed by electrophoresis using 1.5% (w/v) agarose gel. Gels were analyzed using an ultraviolet (UV) transilluminator (UV Tech, France).

### REP- PCR

Genotype comparison was carried out for evaluating clonality of the isolates. The primers REP1 and REP2 (Table 1) were used and PCR amplification of the isolates was performed as described previously [16].

## Results

### Detection of *A. baumannii* in air, surface and water samples

Concentration of airborne bacteria in the hospitals ranged from 1 to 2355 CFU/m^3^. *A. baumannii* was detected in a concentration from 8 to 56 CFU/m^3^ in air samples. The mean concentrations of detected airborne bacteria and *A. baumannii* in each hospital ward are presented in Table 2.

**Table 2:** Mean concentration (CFU/m^3^) of airborne bacteria *(A. baumannii*)^a^ in different hospital wards

	Hospital A	Hospital B	Hospital C	Hospital D
Hospital ward				
ICU	43 (8)	194(14)	23(56)	61 (ND)
OT	617(ND)	16.43 (ND)	28 (ND)	17 (ND)
SW	61(14)	379.63 (ND)	50 (ND)	352 (ND)
IM	32(14)	104 (17)	26 (ND)	19 (ND)

*ICU* intensive care unit, *OT* operating theatre, *SW* surgery ward, *IM* internal medicine ward, *ND* not detected

^a^Concentration of *A. baumannii* in positive samples

*A. baumannii* was detected in 11% (7/64) of air samples. Percent of air positive samples for *A. baumannii* in various wards of four hospitals is presented in Table 3. As the data shows *A. baumannii* was not detected in air samples of hospital D as well as in air samples at any of the operating theatres of hospitals.

**Table 3:** Percentage (No. of positive samples/total samples in each ward) of *A. baumannii* positive environmental samples in different hospital wards

	Hospital A				Hospital B				Hospital C				Hospital D				
Location	ICU	OT	SW	IM	ICU	OT	SW	IM	ICU	OT	SW	IM	ICU	OT	SW	IM	Total
Air sample	25% (1/4)	ND	25% (1/4)	25% (1/4)	25% (1/4)	ND	ND	50% (2/4)	25% (1/4)	ND	ND	ND	ND	ND	ND	ND	11% (7/64)
Surface sample	67% (2/3)	-	33% (1/3)	ND	100% (3/3)	-	ND	ND	ND	-	ND	ND	ND	-	ND	25% (1/4)	17% (7/42)
Water sample	ND	-	ND	ND	ND	-	25% (1/4)	ND	ND	-	ND	ND	ND	-	ND	ND	2% (1/42)

*ICU* intensive care unit, *OT* operating theatre, *SW* surgery ward, *IM* internal medicine ward, *ND* not detected

Table 3 also shows the percent of surface and water samples which *A. baumannii* was detected. *A. baumannii* was detected in 17% (7/42) and 2% (1/42) surface and water samples, respectively. Data of Table 3 shows that *A. baumannii* was detected with the highest frequency in air and surface samples of ICUs.

A total of 40 *A. baumannii* isolates were recovered from positive samples including air samples 30% (12/40), patient beds 67.5% (27/40) and water samples 2.5% (1/40).

### Antimicrobial susceptibility of *A. baumannii* isolates

Analysis of antimicrobial susceptibility in this study showed that 100% (12/12) of *A. baumannii* isolates from air samples were resistant to ceftazidime, imipenem and gentamicin. In other words, all air isolates were multidrug resistant*. A. baumannii* isolated from water sample was also resistant to ceftazidime and gentamicin but not imipenem. However, 70% (19/27) of the isolates from surface samples were resistant to gentamicin and 81% (22/27) and 89% (24/27) were resistant to imipenem and ceftazidime, respectively. Overall, ceftazidime resistant *A. baumannii* was the most frequently detected isolates (92.5%, 37/40) followed by imipeneme resistant (85%, 34/40) isolates.

### OXA-type carbapenemase-encoding genes in *A. baumannii* isolates

**Fig. 1:**
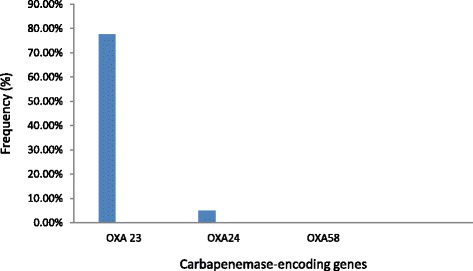
Frequency of detection of different groups of carbapenemase-encoding genes in *A. baumannii* isolates

Figure 1 shows the frequency of carbapenemase-encoding genes in *A. baumannii* isolates. 77.5% (31/40) strains contained OXA-23, 5% (2/40) strains contained OXA-24 and OXA-58 was not detected in any of the strains.

### REP-PCR

**Fig. 2:**
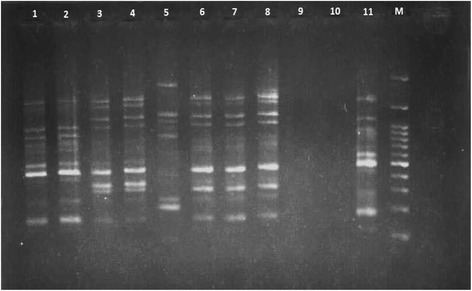
REP-PCR pattern of some *A. baumannii* isolates. 1–8: *A. baumannii* isolates, 9–10: negative control, 11: positive control (*A. baumannii*, ATCC19606), M: 100 bp marker

Analysis of REP-PCR showed 10 different patterns. Figure 2 shows the REP-PCR pattern of some isolates.

## Discussion

Over the past two decades, antibiotic resistant *A. baumannii* has emerged as one of the most problematic hospital acquired pathogens [4, 17, 18].

The results of this study showed the presence of *A. baumannii* in various hospital environments including air, inanimate surface and water. *A. baumannii* was isolated from 11% (7/64) of air samples. Other investigations on hospital air also reported the presence of *Acinetobacter* species and *A. baumannii* [12–14, 19]. In the study of Gao et al. [12] on air samples from burn wards of a general hospital in China, 16 samples were found positive for *A. baumannii*. However, the majority of air samples were negative. Munoz-Price et al. [14] also reported the presence of *A. baumannii* in 22.6% (12/53) of air samples in a trauma ICU. Our results showed that the highest detection was related to ICUs. It has been demonstrated that *A. baumannii* is responsible for a high percentage of ventilator-associated pneumonia which occurs predominantly in ICU patients with mechanical ventilation [1, 4]. There is some evidence that hospital air plays a significant role in the transmission of *A. baumannii* infections [2, 11]. Bernards et al. [20] reported the airborne route of *A. baumannii* outbreaks in two Dutch hospitals. *A. baumannii* aerosols could be released from various sources including respiratory droplets produced by patients, ventilation and air conditioning systems and also ward activities such as those generated by bed making and mechanical floor cleaning [2, 13, 21].

Analysis of water samples revealed the presence of *A. baumannii* in one sample of SW. Hospital water systems were known to be colonized by some nosocomial pathogens such as *Legionella pneumophila* and *Pseudomonas aeruginosa* and could act as a potential source for aerosolized nosocomial pathogens [22, 23].

It is also possible that airborne bacteria deposited on inanimate surfaces [2]. Our results showed that 17% (7/42) of patient beds were contaminated with *A. baumannii* and ICUs had the highest rate of contamination. Study of Custovic et al. [4] showed that 17.7% (31/175) swabs were taken from hospital surfaces, medical equipment and hands of medical staffs were positive for some nosocomial pathogens with the highest isolation rate of *A. baumannii* (51.6%).

Detection of *A. baumannii* in air, water and inanimate surface samples revealed that hospital environments could act as a potential route for transmission of *A. baumannii* infections especially in ICUs. Several studies demonstrated the relation between *A. baumannii* outbreaks and environmental sources such as patient beds, air conditioners and ventilation equipment. In the study of Aygun et al. [24] *A. baumannii* was isolated from 39.3% (22/59) environmental samples obtained by swabbing in ICU. They concluded that environmental contamination has an important reservoir role in outbreaks of *A. baumannii* in ICUs. Results of Tena et al. [25] showed that all five clinical isolates and one environmental isolate belonged to a single clone. Based on the clonal relationship of the isolates by pulsed-field gel electrophoresis (PFGE), they concluded that the infection source has probably been the hands of the healthcare workers [25]. Similarly, Cicek et al. [7] suggested that all the patients and environmental isolates were derived from a common source.

*A. baumannii* isolates showed the highest resistance towards ceftazidime (92.5%, 37/40). It has been reported that a high portion of clinically isolated *A. baumannii* are resistant to cephalosporins such as ceftazidime [18]. Our results also showed that a high percentage of *A. baumannii* isolates were resistant to imipenem (85%, 34/40) and all air isolates were multidrug resistant [6]. In consistent with our results, Gao et al. [12] reported that 93.75% (15/16) *A. baumannii* isolates from air samples were resistant to imipenem. Carbapenems resistant strains of *A. baumannii* have been associated with considerable mortality and hospital costs [26, 27]. Crude mortality of infections caused by these strains ranges from 16 to 76% [27]. High resistance rate of *A. baumannii* to carbapenems has been frequently observed in clinical isolates [4, 28]. However, some European studies reported much lower resistance to carbapenems [28]. Antimicrobial susceptibility analysis revealed the lowest resistance towards gentamicin.

However, 67.5% (27/40) of *A. baumannii* isolates were multidrug resistant [6]. Multidrug resistant *A. baumannii*, in particular carbapenem resistant has a propensity to cause hospital infections [18].

The results showed that OXA-23 was the most frequent gene (77.5%, 31/40) detected in *A. baumannii* isolates. The OXA-23 gene was detected in all of air isolates except one isolate. This result suggests that the OXA-23 was the main cause of the resistance of *A. baumannii* isolates from air, water and surface samples in our hospitals. Other investigations also reported OXA-23 group as the most prevalent carbapenemase-encoding gene [18]. Gao et al. [12] also found 15/16 strains from air samples were positive for OXA-23 gene. Pajand et al. [10] reported a high prevalence of blaOXA-23 (68%) in carbapenems resistant *A. baumannii* isolates. However, we could not detect any OXA-58 gene and OXA-24 was found only in two isolates of air samples. In the study of Gao et al. [12], OXA-24 and −58 were not detected in any air isolates. Tena et al. [25] detected OXA-24 carbapenemase in all five isolates from patients and one isolate from surface of a serum container. Conversely, studies in Italy reported the frequent isolation of OXA-58 producing *A. baumannii* [29].

Genotype comparison of the 40 isolates showed 10 distinct patterns. REP-PCR results analysis showed no high similarity between air, surface and water isolates from various wards. A unique REP-PCR profile was only observed in isolates from a surface sample of SW in hospital A and air sample isolate of ICU in hospital C. These results demonstrated that *A. baumannii* isolates were derived from various sources in hospital environments.

## Conclusion

The results of this study showed the presence of multidrug resistant *A. baumannii* in various hospital environments including air, water and surface. Based on the results of PCR analysis of carbapenemase-encoding genes, OXA-23 was the main cause of the antibiotic resistance of *A. baumannii* isolates. Therefore, early detection and implementation of appropriate control measures are crucial in preventing of transmission of *A. baumannii* infections through hospital environments, especially in ICUs.

## Abbreviations

*A. baumannii*: *Acinetobacter baumannii*
AGI: All-glass impinge
CFU: Colony forming unit
ICU: Intensive care unit
IM: Internal medicine
OT: Operating theatre
PCR: Polymerase chain reaction
REP-PCR: Repetitive extragenic palindromic sequences PCR
SW: Surgery ward
